# Integration of multiple-omics data to reveal the shared genetic architecture of educational attainment, intelligence, cognitive performance, and Alzheimer’s disease

**DOI:** 10.3389/fgene.2023.1243879

**Published:** 2023-10-12

**Authors:** Fuxu Wang, Haoyan Wang, Ye Yuan, Bing Han, Shizheng Qiu, Yang Hu, Tianyi Zang

**Affiliations:** ^1^ Center for Bioinformatics, Faculty of Computing, Harbin Institute of Technology, Harbin, Heilongjiang, China; ^2^ Beidahuang Industry Group General Hospital, Harbin, China; ^3^ Aier Eye Hospital, Harbin, China

**Keywords:** Alzheimer’s disease, educational attainment, intelligence, cognitive performance, Mendelian randomization, genome-wide association studies, conditional false discovery rate

## Abstract

Growing evidence suggests the effect of educational attainment (EA) on Alzheimer’s disease (AD), but less is known about the shared genetic architecture between them. Here, leveraging genome-wide association studies (GWAS) for AD (N = 21,982/41,944), EA (N = 1,131,881), cognitive performance (N = 257,828), and intelligence (N = 78,308), we investigated their causal association with the linkage disequilibrium score (LDSC) and Mendelian randomization and their shared loci with the conjunctional false discovery rate (conjFDR), transcriptome-wide association studies (TWAS), and colocalization. We observed significant genetic correlations of EA (r_g_ = −0.22, *p* = 5.07E-05), cognitive performance (r_g_ = −0.27, *p* = 2.44E-05), and intelligence (r_g_ = −0.30, *p* = 3.00E-04) with AD, and a causal relationship between EA and AD (OR = 0.74, 95% CI: 0.58–0.94, *p* = 0.013). We identified 13 shared loci at conjFDR <0.01, of which five were novel, and prioritized three causal genes. These findings inform early prevention strategies for AD.

## Introduction

Alzheimer’s disease (AD) remains the most common neurodegenerative disease among the elderly, which affects more than 44 million people worldwide (Scheltens et al., 2016; Van Cauwenberghe et al., 2016). Known drugs or treatments may hardly completely reverse the progression of AD, so understanding the modifiable risk factors of AD remains the first choice for prevention.

Educational attainment (EA) and cognitive performance are known modifiable factors for dementia (Larsson et al., 2017; Alzheimer’s Association, 2020). Years of continuous formal education and cognitive training make brains form a cognitive reserve (Stern, 2012). Brains of individuals with higher education would continue to perform cognitive tasks even if the excessive accumulation of amyloid-beta (Aβ) and tau protein exists (Stern et al., 1994; Dekhtyar et al., 2019). Amieva et al. (2014) followed up 171 less-educated people and 271 highly educated people for 20 years and found that cognitive performance of highly educated people decreased 15–16 years before reaching the threshold of AD, while the less-educated people developed AD in only 7 years. In other words, EA greatly delays the progression of AD by maintaining cognitive performance.

Significantly, EA has been found to have associations with both intelligence and cognitive performance. Intelligence can continuously improve through learning, and individuals with higher intelligence or childhood intelligence tend to have longer years of schooling, as evidenced by previous studies (Anderson et al., 2020; Lovden et al., 2020). Moreover, epidemiological research has indicated that lower IQ in children can be a predictor of poor cognitive function and an increased risk of developing AD later in life (Snowdon et al., 1996; Vinueza-Veloz et al., 2020). Although there is currently no direct evidence establishing a link between early intelligence and the degree or type of neuropathological features of dementia in older adults, it is possible that the phenotypic association between the two is influenced by shared genetic variants (Yeo et al., 2011). Consequently, the correlation between these cognition-related phenotypes (EA, cognitive performance, intelligence, and childhood intelligence) may introduce some complexity when discerning their causal relationship with AD. Furthermore, the shared genetic architecture and causal genes underlying the cognition-related phenotypes and AD remain unidentified.

**FIGURE 1:**
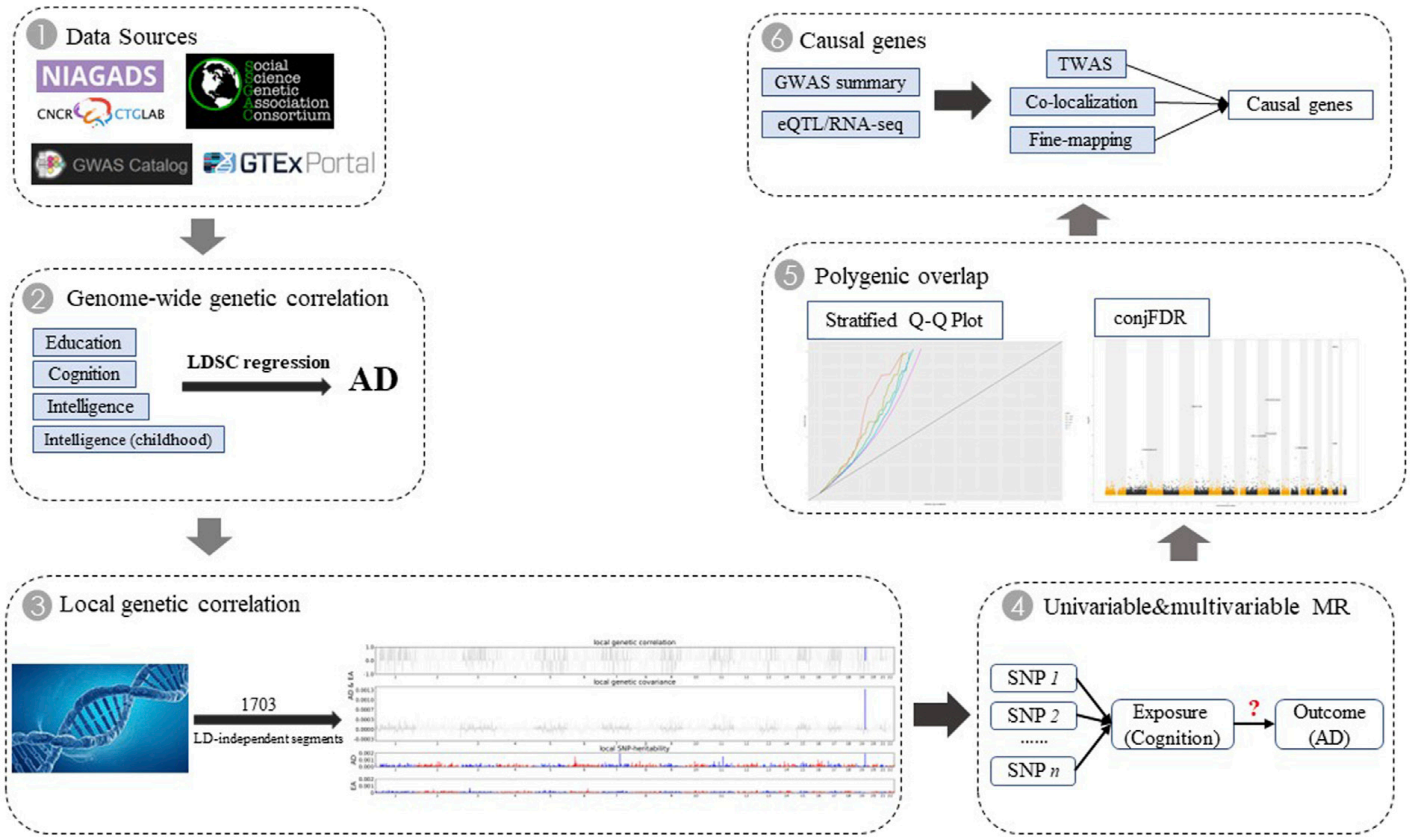
Overall study design. We first retrieved five GWAS data sources. For cognition-related phenotypes that showed significant genetic correlation with AD, we conducted further genome-wide cross-trait analysis to investigate genetic overlap between them by integrating multiple-omics data.

Although observational studies are difficult to adjust for these complex covariates, Mendelian randomization (MR) studies based on genome-wide association studies (GWAS) may have the power to provide an independent effect of each exposure on AD. In this study, we aimed to investigate the shared genetic architecture between AD and four cognition-related phenotypes, including genetic correlations, local genetic correlations, independent causal relationship, polygenic overlap, and shared causal genes (Figure 1). We explored the underlying potential biological mechanisms and provided an important contribution to prevent the occurrence of AD.

## Methods

### Participant samples

We obtained GWAS summary statistics for AD from a meta-analysis of 46 AD GWAS datasets (21,982 cases and 41,944 controls) by the International Genomics of Alzheimer’s Project (IGAP) (Lambert et al., 2013; Kunkle et al., 2019). A total of 9,456,058 common variants and 2,024,574 rare variants passed the quality control (Kunkle et al., 2019). We obtained GWAS summary statistics for EA and cognitive performance from a meta-analysis of 71 cohort-level results by the Social Science Genetic Association Consortium (SSGAC), containing 1,131,881 and 257,828 individuals, respectively (Lee et al., 2018). GWAS summary statistics for intelligence were obtained from the Center for Neurogenomics and Cognitive Research (CNCR) CTGlab (Sniekers et al., 2017). Sniekers et al. (2017) performed a meta-analysis of several intelligence GWAS, including UK Biobank web-based measure (N = 17,862), UK Biobank touchscreen measure (N = 36,257), Childhood Intelligence Consortium (CHIC) (N = 12,441), and five additional cohorts (N = 11,748). We obtained childhood intelligence GWAS from CHIC (Benyamin et al., 2014). CHIC assessed the intelligence of 17,989 children of European origin aged 6–18 years and performed the single-nucleotide polymorphism (SNP) classification of GWAS, including six discovery (N = 12,441) and three replication (N = 5,548) cohorts (Benyamin et al., 2014). Details of the participants were shown in the original studies (Lambert et al., 2013; Sniekers et al., 2017; Lee et al., 2018; Kunkle et al., 2019).

We removed the rare variants (MAF <0.01) and performed the exchange of the reference genome (hg18/hg19) required in part of the study. We only focused on autosomal chromosomes and excluded the HLA region in this study. All participants were of European descent, and informed consent was obtained from all the participants in each study (Sniekers et al., 2017; Lee et al., 2018; Kunkle et al., 2019). The study protocols were approved by the corresponding institutional review boards.

### Genome-wide genetic correlation analysis

We performed a cross-trait linkage disequilibrium score (LDSC) regression to evaluate the genetic correlations of AD with the four cognition-related phenotypes (Bulik-Sullivan B. et al., 2015; Qiu et al., 2023). We used precomputed LD scores derived from HapMap3 reference panels, which contained more than one million European participants from UK Biobank (Bulik-Sullivan B. et al., 2015; Bulik-Sullivan B. K. et al., 2015). LDSC regression first calculated the average LD between SNPs across the genome and then regression of the GWAS summary statistics based on baseline LD scores (Bulik-Sullivan B. et al., 2015). The slope of the regression line represented an estimate of heritability for a trait or disease. Notably, LDSC also corrected for confounding factors such as sample overlap and population stratification, which might affect heritability estimates. More details about the LDSC algorithm have been reported in previous studies (Bulik-Sullivan B. et al., 2015). The statistically significant association after multiple testing is defined to be *p* < 0.05/4 = 0.0125.

### Local genetic correlation analysis

We divided the gene components into pre-specified LD-independent segments (1,703 segments) and calculated the local genetic correlation of each segment separately. Herein, we performed two powerful computing tools: Heritability Estimation from Summary Statistics (HESS) and pairwise analysis of GWAS (GWAS-PW) (Pickrell et al., 2016; Shi et al., 2017). HESS was used for estimating and visualizing the local SNP-heritability and genetic correlations and calculating genetic covariance to measure the similarity between a pair of traits driven by genetic variants (Shi et al., 2017). GWAS-PW was used for evaluating the local correlations and associated SNPs of each segment, under a Bayesian colocalization framework (Pickrell et al., 2016; Shi et al., 2017). The statistically significant association for HESS is defined to be *p* < 0.05/1703 = 2.94E-05 after correcting for multiple testing. The statistically significant association for GWAS-PW is defined to be posterior probability 3 (PPA_3) > 0.9.

### Univariable MR

MR study is an effective method for the analysis of causal inference in epidemiology (Hemani et al., 2018). MR analysis uses independent genome-wide significant SNPs (*P* < 5E-08, *r* ^2^ < 0.3) as instrumental variables (IVs) to estimate the causal estimates of exposure on the outcome (Hemani et al., 2018; Qiu et al., 2021; Hu et al., 2022; Qiu et al., 2022). The genotypes of SNP instruments are established at birth and may not be altered by confounding factors. Here, we selected 262, 116, and 146 independent genome-wide significant SNPs from GWAS summary statistics for EA, cognitive performance, and intelligence, respectively, as instruments to perform two-sample MR (Hemani et al., 2018). We implemented MR-Egger and MR Pleiotropy RESidual Sum and Outlier (MR-PRESSO) as sensitivity analyses (Bowden et al., 2016; Hemani et al., 2018; Verbanck et al., 2018). The TwoSampleMR (version 0.5.6) and MR-PRESSO (version 1.0) R packages were used for MR analyses. *p* < 0.05/3 = 0.0167 was considered significant enrichment after multiple testing.

### mtCOJO method and GSMR analysis

The association across EA, cognitive performance, childhood intelligence, and intelligence may interfere with the effect of one of these cognition-related phenotypes on AD. Here, we performed multi-trait-based conditional and joint (mtCOJO) analysis to adjust for pleiotropic SNPs among the cognition-related phenotypes and then implemented generalized summary-data-based Mendelian randomization (GSMR) analysis using conditional GWAS data (Zhu et al., 2018).

The mtCOJO method is performed as described in Zhu et al. (2018). If we adjust for the three covariates (cognitive performance, childhood intelligence, and intelligence) when estimating the influence of an SNP on EA, it is to use the causal estimates of cognitive performance, childhood intelligence, and intelligence on EA calculated by GSMR analysis as the condition in a GWAS conditional analysis (Zhu et al., 2018). In other words, in exploring the causal relationship between EA and AD, we removed the effects of three covariates from exposure. As a result, we obtained conditional GWAS data for each phenotype after adjusting for pleiotropy.

Leveraging conditional GWAS data, we investigated the causality between cognition-related phenotypes and AD using GSMR analysis (Hemani et al., 2018). Based on the MR framework, GSMR performs summary-based Mendelian randomization (SMR) analysis on each SNP instrument separately, considers the sampling variance of each SNP and LD between SNPs, and integrates the causal estimation of all SNP instruments through the generalized least squares method (Zhu et al., 2016; Zhu et al., 2018). Thus, GSMR excluded the estimation biases that might arise from pleiotropy (Zhu et al., 2018). We then carried out an instrument selector called heterogeneity in dependent instruments (HEIDI)-outlier to distinguish causality from pleiotropy (Zhu et al., 2018). Finally, we used 1000 Genomes Phase III as LD reference panels to clump the SNPs and selected independent genome-wide significant SNPs of conditional GWAS (LD: r < 0.1, *p* < 5E-08).

### Conditional Q–Q plots

We performed genomic controls to adjust for the expansion and deflation of the empirical null distribution in GWAS due to population stratification and overcorrection of test statistics for polygenic traits (Yang et al., 2011; Andreassen et al., 2013). To assess the pleiotropic enrichment and shared risk loci of cognition-related phenotypes associated with AD, we generated conditional quantile–quantile (Q–Q) plots and computed the conditional false discovery rate (condFDR) statistics. Conditional Q–Q plots were generated using the log_10_(*p*) value of all SNP loci for the main trait (e.g., AD) and the log_10_(*p*) values of SNP loci across several different thresholds for the conditional trait (e.g., EA) (Andreassen et al., 2013; Andreassen et al., 2014; Desikan et al., 2015). The thresholds of condFDR included *p* < 1, *p* < 0.1, *p* < 0.01, *p* < 0.001, and *p* < 0.0001. Details of these statistical methods have been described in previous publications (Andreassen et al., 2013; Andreassen et al., 2014; Desikan et al., 2015).

### Conditional false discovery rate

The unconditional FDR (uFDR) refers to the probability that an SNP locus is associated with a trait as a false positive and is expressed as the ratio of the observed *p*-value to the observed quantile under the null hypothesis (Andreassen et al., 2013; Andreassen et al., 2014; Desikan et al., 2015). The condFDR is an extension of uFDR defined as the probability that an SNP locus is not associated with a main trait *i* (e.g., AD) if the *p*-value in both traits is less than a preset significance threshold and *vice versa* (Andreassen et al., 2013; Andreassen et al., 2014; Desikan et al., 2015). The conjFDR minimizes the effect of a single trait driving the shared association signal (Andreassen et al., 2013; Andreassen et al., 2014; Desikan et al., 2015). When the conjFDR value of this SNP is less than 0.01, it is generally considered to be significantly associated with both traits. The conjFDR method is implemented using the R package “GWAScFDR.”

### eQTL analysis and functional enrichment

To further assess whether the shared risk loci of AD and cognition-related phenotypes could regulate gene expression, we performed the expression quantitative trait loci (eQTL) analysis in whole blood and 13 brain tissues (brain amygdala, anterior cingulate cortex, caudate basal ganglia, cerebellar hemisphere, cerebellum, cortex, hippocampus, hypothalamus, frontal cortex, nucleus accumbens basal ganglia, putamen basal ganglia, spinal cord cervical, and substantia nigra) from the Genotype-Tissue Expression (GTEx) (GTEx Consortium et al., 2017). 1E-06 was used as the threshold for FDR <0.05. To explore the functional locations and pathways of shared risk loci, we performed enrichment analysis in Gene Ontology (GO) and Kyoto Encyclopedia of Genes and Genomes (KEGG) using the clusterProfiler package (Yu et al., 2012). *p* < 0.05 was considered significant enrichment after multiple testing.

### TWAS and colocalization analysis

GWAS identifies susceptibility loci for complex traits, but whether these loci influence the phenotypes through gene expression remains unknown. TWAS were used as an association test to identify the expression of putative risk genes in complex traits (Gusev et al., 2016). Here, we selected dorsolateral prefrontal cortex (DLPFC) RNA-seq datasets from the CommonMind Consortium (CMC) as the expression reference weights and imputed expression into GWAS summary statistics using Fusion software (Fromer et al., 2016; Gusev et al., 2016; Senthil et al., 2017). For genes showing significant association in the TWAS test (*p* < 0.05/No. of genes), we further performed colocalization analysis to scan for shared causal genes (Giambartolomei et al., 2014; Wallace, 2020). Colocalization used the Bayesian statistical test to calculate the posterior probability of five hypotheses (H0–H5), and the posterior probability PPH4 > 0.75 was interpreted as colocalization generally. The common significance of TWAS and colocalization analysis ensured the accuracy of the association test (Gusev et al., 2016).

### Fine-mapping of causal gene sets

In order to confirm the credibility of the causal genes identified by TWAS/colocalization analysis, we carried out the fine-mapping of causal gene sets (FOCUS) (Mancuso et al., 2019). FOCUS assigned the probability of associated signals to the risk genes identified by TWAS, which could be used for gene prioritization in functional analysis (Mancuso et al., 2019). The overlapping signals of TWAS, colocalization, and fine-mapping might be credible causal genes. We prioritized the mapping in brain tissues and showed the predicted expression correlation of each gene within the risk region.

## Results

### Shared genomic architectures

We performed a cross-trait LDSC regression and observed negative genetic correlations of AD with EA (r_g_ = −0.22, *p* = 5.07E-05), cognitive performance (r_g_ = −0.27, *p* = 2.44E-05), and intelligence (r_g_ = −0.30, *p* = 3.00E-04) (Table 1). Nevertheless, we found non-significant genetic correlations of childhood intelligence and AD (r_g_ = −0.02, *p* = 0.60) (Table 1).

**TABLE 1:** Genetic correlations between AD and cognition-related phenotypes.

Phenotype	*r*_*g*_	*r*_*g*_ *_se*	*p*
Educational attainment	−0.218	0.054	5.07E-05
Cognitive performance	−0.269	0.064	2.44E-05
Childhood intelligence	−0.017	0.033	0.603
Intelligence	−0.304	0.085	3.00E-04

*r*_*g*_: genetic correlation, *r*_*g*_ *_se*: standard error of genetic correlation, *r*_*g*_ *_z*: Z-score of genetic correlation.

*p*: the statistically significant association is defined to be *p* < 0.05/4 = 0.125.

**FIGURE 2:**
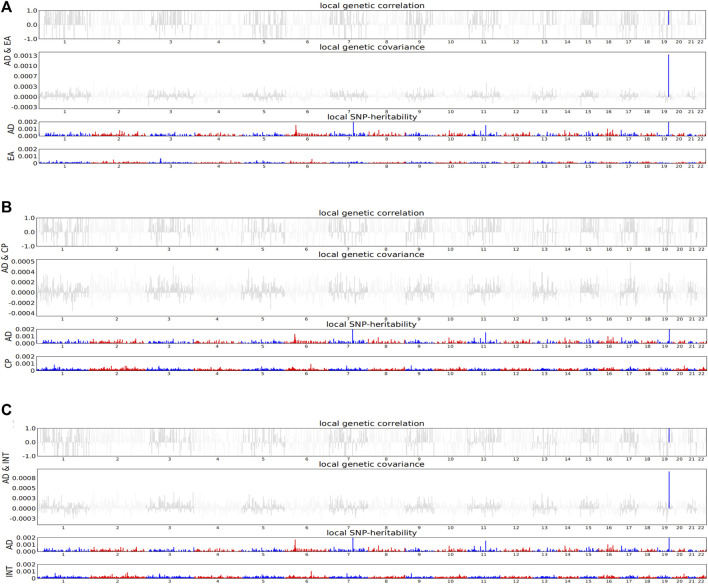
HESS analysis of AD with EA, cognitive performance, and intelligence. For each sub-figure, top and middle parts represent local genetic correlation and covariance, respectively, and colored bars represent loci that have significant local genetic correlation and covariance. Bottom part represents local SNP-heritability for individual traits, and colored bars represent loci that have significant local SNP-heritability. *AD*, Alzheimer’s disease; *EA*, educational attainment; *CP*, cognitive performance; *INT*, intelligence. **(A)** The local genetic correlation of AD and EA. **(B)** The local genetic correlation of AD and cognitive performance. **(C)** The local genetic correlation of AD and intelligence.

In order to observe the similarity between two traits driven by genetic variants in specific regions of the genome, we calculated the local genetic covariance of AD with EA, cognitive performance, and intelligence in 1,703 regions with independent LD. As a result, both EA (*P*_*AD&EA*_ = 1.62E-09) and intelligence (*P*_*AD&intelligence*_ = 4.33E-06) were associated with AD in the chromosome 19: 44.7M–46.1M region (Figure 2, Supplementary Tables S1–3). The local genetic overlap of AD and EA was also shown in the chromosome 14: 9.12M–9.31M region using GWAS-PW analysis (Supplementary Tables S4–6).

### Independent causal relationship

**FIGURE 3:**
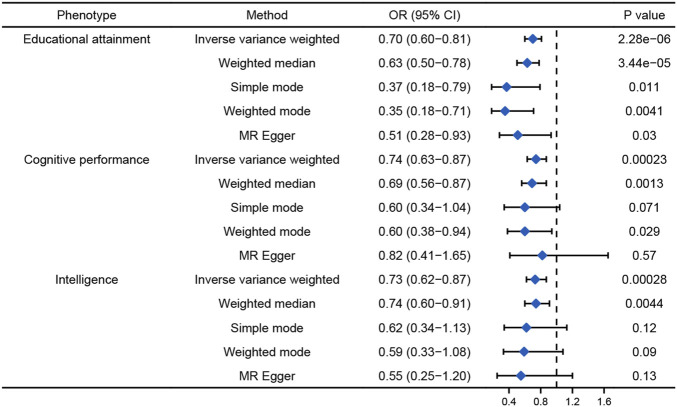
Univariable MR results for the causal relationship between cognition-related phenotypes and AD.

Univariable MR analysis showed causal relationships between the genetically predicted cognition-related phenotypes and AD (Figure 3). Every one standard deviation (SD) increase in EA, cognitive performance, and intelligence was associated with 30%, 26%, and 27% lower risk of AD (EA: odds ratio (OR), 0.70; 95% confidence interval (CI), 0.60 to 0.81, *p* = 2.28E-06; cognitive performance: OR, 0.74; 95% CI, 0.63 to 0.87, *p* = 0.00023; and intelligence: OR, 0.73; 95% CI, 0.62 to 0.87, *p* = 0.00028), respectively. MR-PRESSO and MR-Egger found no evidence of directional pleiotropy.

Owing to the potential causal relationship between EA and AD and the possible limitations of multivariable MR, we further performed mtCOJO analysis to adjust for pleiotropic SNPs among the cognition-related phenotypes and then implemented GSMR analysis using conditional GWAS (Zhu et al., 2018). After conditioning, genetically predicted EA was significantly associated with AD risk (OR = 0.74, 95% CI: 0.58–0.94, *p* = 0.013) (Supplementary Table S7). However, there was a non-significant causal relationship between cognitive performance or intelligence and AD. The number of SNP instruments for childhood intelligence was not sufficient to perform MR.

### Pleiotropic enrichment and polygenic overlap

**FIGURE 4:**
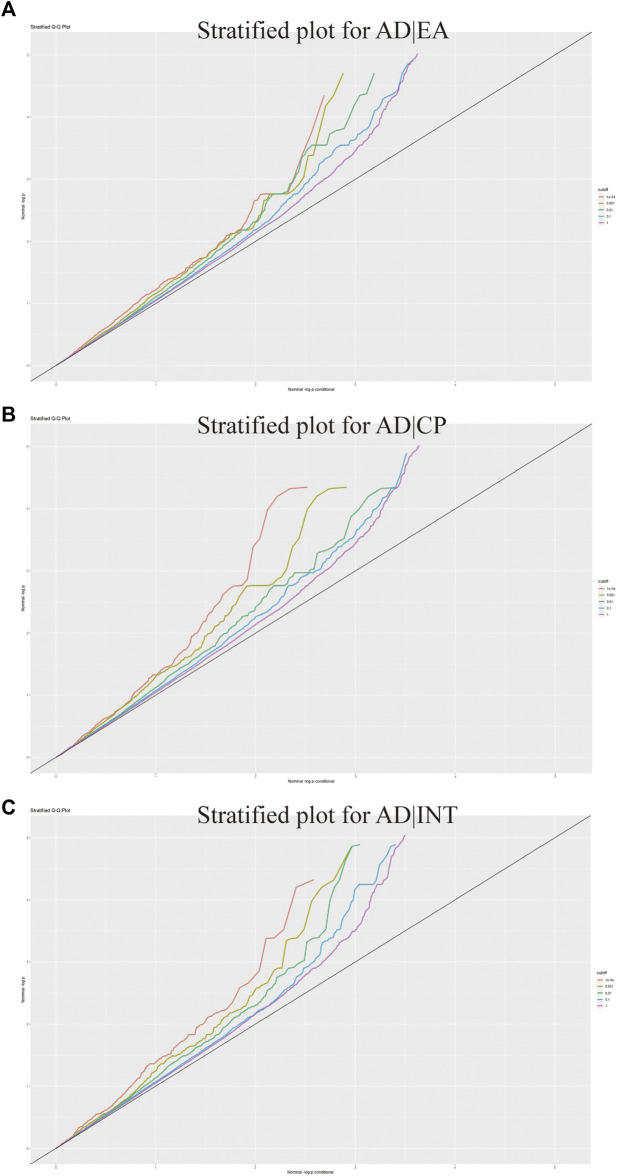
Conditional quantile–quantile plots. Dotted lines indicate the expected line under the null hypothesis, and leftward deflection demonstrates the degree of pleiotropic enrichment. **(A)** Conditional Q-Q plots for AD and EA. **(B)** Conditional Q-Q plots for AD and cognitive performance. **(C)** Conditional Q-Q plots for AD and intelligence.

To describe the pleiotropic enrichment between cognition-related phenotypes and AD, we generated conditional Q–Q plots for conditioning the cognition-related phenotypes on AD (Andreassen et al., 2013; Andreassen et al., 2014; Desikan et al., 2015). A significant upward deflection of the conditional Q–Q plot was observed for EA, cognitive performance, and intelligence as conditional traits, suggesting a significant pleiotropic enrichment of AD with the cognition-related phenotypes (Figure 4).

**FIGURE 5:**
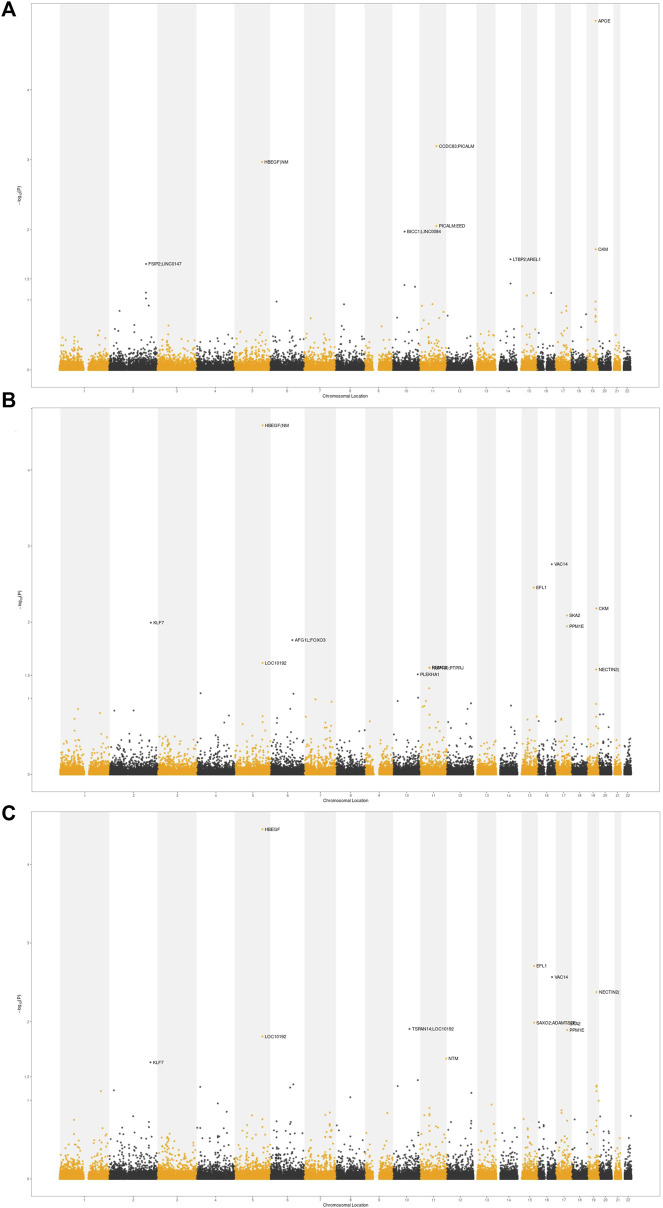
Conditional Manhattan plot. The shared risk loci between AD and cognition-related phenotypes were marked. The statistically significant causality is defined to be conjFDR <0.05. **(A)** AD and EA. **(B)** AD and cognitive performance. **(C)** AD and intelligence.

In order to identify the overlapping loci of cognition-related phenotypes and AD, we applied a conjFDR statistical framework based on GWAS. At conjFDR <0.01, we identified 4, 5, and 4 shared risk loci for AD and EA, cognitive performance, and intelligence, respectively (Figure 5; Table 2). Among these shared loci, *APOE*, *PICALM*, and *HBEGF* were susceptibility loci reported in previous GWAS for AD (Naj et al., 2014; Rosenberg et al., 2016; Jun et al., 2017). *VAC14*, *EFL1*, *CKM*, *SKA2*, and *NECTIN2* were novel risk loci. *HBEGF* was identified in the shared risk loci for both AD and all cognition-related phenotypes. *HBEGF* encodes a growth factor called heparin-binding epidermal growth factor-like growth factor (HB-EGF), which binds to APP, the transmembrane glycoprotein central to AD, and acts synergistically with EGF to promote ERK signaling and neuritogenesis (da Rocha et al., 2021).

**TABLE 2:** Shared risk loci of AD and cognition-related phenotypes using conjFDR.

Trait	SNP	Role	Gene	Position	conjFDR
Educational attainment	rs405509	Upstream	*APOE*	chr19: 45,408,836	0.000010
	rs598561	Intergenic	*CCDC83* and *PICALM*	chr11: 85,652,826	0.00064
	rs7268	3′-UTR	*HBEGF*	chr5: 139,712,550	0.0011
	rs3844143	Intergenic	*PICALM* and *EED*	chr11: 85,850,243	0.0088
Cognitive performance	rs7268	3′-UTR	*HBEGF*	chr5:139,712,550	2.60E-05
	rs11649476	Intronic	*VAC14*	chr16:70,736,752	0.0017
	rs2665103	Intronic	*EFL1*	chr15:82,432,715	0.0035
	rs344816	Intronic	*CKM*	chr19:45,825,626	0.0066
	rs12051897	Intronic	*SKA2*	chr17:57,207,540	0.0082
Intelligence	rs2074613	Intronic	*HBEGF*	chr5:139,714,564	3.57E-05
	rs2665103	Intronic	*EFL1*	chr15:82,432,715	0.0020
	rs11649476	Intronic	*VAC14*	chr16:70,736,752	0.0027
	rs6859	3′-UTR	*NECTIN2*	chr19:4,5,382,034	0.0042

conjFDR, conjunctional false discovery rate.

We further evaluated the cis-expression of these loci in GTEx and found that they regulated the expression of *WDR55*, *RP11-394B2.1*, *AP3B2*, *SAXO2*, *ADAMTS7P1*, *UBE2Q2P2*, *GOLGA2P10*, *GOLGA6L9*, *MARK4*, *KLC3*, *TRIM37*, *RAD51C*, *TEX14*, *AC099850.1*, *SKA2*, *PRR11*, and *NECTIN2* in whole blood and various brain tissues (Supplementary Table S8). In addition, we enriched these risk loci in GO and KEGG. The shared risk loci of EA and AD were mainly enriched in the formation and regulation of Aβ and the binding of tau protein or lipoprotein receptor (Supplementary Figure S1). The shared risk loci of cognitive performance and AD were mainly enriched in keratinocyte migration and glycoprotein biosynthetic processes (Supplementary Figure S2).

### Credible causal genes

Shared loci identified by conjFDR (closest to risk SNPs at physical distance) may still be affected by limitations of GWAS (Zhu et al., 2016). In order to identify the credible causal genes of these phenotypes, we conducted TWAS, colocalization, and fine-mapping. We selected brain tissue RNA-seq datasets as expression reference weights and performed the expression imputation to GWAS on each chromosome in turn (Gusev et al., 2016). As a result, a total of 18 AD genes, 439 EA genes, 292 cognitive performance genes, and 276 intelligence genes showed strong associations with the brain (Supplementary Tables S9–12). *TSPAN14*, *FAM180B*, *GOLGA6L9*, and *MTCH2* showed significant correlation signals in both AD and cognitive performance. *TSPAN14*, *FAM180B*, and *GOLGA6L9* showed significant correlation signals in both AD and intelligence. Almost all causal genes identified by TWAS were replicated in colocalization (Supplementary Tables S9–12). Fine-mapping prioritized three credible causal genes (*TSPAN14*, *FAM180B*, and *GOLGA6L9*), which were selected in the credible set the most times in AD and cognition-related phenotypes (Supplementary Table S13).

## Discussion

In this study, we observed substantial polygenic overlap between cognition-related phenotypes and AD. We found high genetic correlations of AD with EA, cognitive performance, and intelligence but without childhood intelligence. Similarly, previous studies showed that genetic variants that mediated the biological effects of AD were unlikely to operate early in life, and no evidence showed that the genetic burden of AD was linked to early cognition (Korologou-Linden et al., 2019; Lamballais et al., 2020). As years of education increased, intelligence might increase and become a protective factor for AD. Further local genetic correlation analysis identified chromosome 19: 44.7M–46.1M as a significantly associated region for EA, intelligence, and AD. Most of the genes in this region were associated with AD risk, such as *APOE*, *TOMM40*, *PVRL2*, *APOC1*, and *MARK4* (Zhou et al., 2019; Serrano-Pozo et al., 2021).

Given the interference of covariates among the four cognition-related phenotypes, we used two approaches to investigate the independent causal relationship: multivariable MR and mtCOJO analysis. The results suggested that higher EA might causally reduce the risk of AD, independently of cognitive performance and intelligence. Similarly, Korologou-Linden et al. (2019) found that when SNPs related to education were removed, the association between low learning performance of adolescents and AD was weakened. Most of these behavioral cognition/education-related phenotypes had ambiguous bidirectional causality. Considering the results of our study alone, we speculated that intelligence would change significantly with the increase in age, years of education, and cognitive training and that the acquired effect was the real reason for AD (Polderman et al., 2015).

Using conditional Q–Q plots and conjFDR statistical framework, we found significant pleiotropic overlap between AD and cognition-related phenotypes and identified 11 shared risk loci: *APOE*, *CCDC83*, *PICALM*, *HBEGF*, *PICALM*, *EED*, *VAC14*, *EFL1*, *CKM*, *SKA2*, and *NECTIN2*. Although *VAC14*, *EFL1*, *CKM*, *SKA2*, and *NECTIN2* were novel risk loci, most of them were reported to be associated with AD, the risk of cognitive decline, or other brain diseases. Given the limitations of traditional GWAS, we further integrated GWAS and eQTL of brain tissues to identify potential causal genes that affected traits by regulating gene expression (Zhu et al., 2016; Tam et al., 2019). Although methods to identify causal genes are still lacking, we assessed the most credible risk genes using three approaches (TWAS, colocalization, and fine-mapping) that approximate causal genes. In this way, we extended the risk loci of GWAS to the transcriptome level and prioritized *TSPAN14*, *FAM180B*, and *GOLGA6L9* as the credible causal genes. Among these genes, *TSPAN14* regulates maturation and trafficking of the transmembrane metalloprotease *ADAM10*, and *ADAM10* is involved in reducing the generation of Aβ peptides (Kunkle et al., 2019; Schwartzentruber et al., 2021). *FAM180B* is also a potential susceptibility gene of AD and appears in the protein–protein interaction network associated with *APOE* (Han et al., 2017). *MTCH2* induces the production of solute carriers, which is reported as a risk gene for AD in multiple brain tissue transcripts (Ruggiero et al., 2017). Although *GOLGA6L9* is a novel locus never reported previously, variant rs2665103, the shared risk loci of AD and cognition-related phenotypes identified by conjFDR statistics, upregulates *GOLGA6L9* expression in cerebellum, suggesting its potential biological role (*β* = 0.56, *p* = 7.4E-08).

Based on large-scale GWAS summary statistics, our study has several strengths. First of all, GWAS for EA (1,131,881 individuals), cognitive performance (257,828 individuals), intelligence (78,308 individuals), and childhood intelligence (17,989 individuals) contain huge sample sizes, which greatly improves statistical power. Second, we adjust the covariates and provide unbiased causal estimation in the study of causality. Third, since it is difficult to determine the true causal genes in the current research, we perform TWAS/colocalization to test the association between risk gene regions and expression and verify the credible causal genes in fine-mapping. However, we acknowledge certain limitations in our study. The 13 cohorts of intelligence GWAS consists of eight children (<18 years; N = 19,509) and five adult cohorts (18–78 years; N = 58,799) (Sniekers et al., 2017). If the childhood intelligence covariates are removed from the intelligence GWAS, the number of remaining SNPs may be inadequate for instrumental variable analysis and genetic association analysis. Finally, the different analyses performed in the study are interconnected and built upon each other to provide a comprehensive understanding of the genetic relationship between AD and cognition-related traits. These findings suggest that implementing early prevention strategies that focus on education and cognitive training could potentially reduce the risk of developing AD. The identification of shared risk loci and credible causal genes provides potential targets and personalized medicine approaches for future therapeutic interventions.

In conclusion, this genome-wide cross-trait analysis strengthened the view that genetically predicted EA, cognitive performance, and intelligence were statistically related to AD risk. We identified 11 pleiotropic risk loci of AD and cognition-related phenotypes, of which five were novel. Our research provided new insights into the shared genetic basis of AD and cognition-related phenotypes from multiple levels and opened a new way for the early prevention of AD.

## Data Availability

The original contributions presented in the study are included in the article/Supplementary Material. Further inquiries can be directed to the corresponding authors.
