# Erratum: Integration of multiple-omics data to reveal the shared genetic architecture of educational attainment, intelligence, cognitive performance, and Alzheimer’s disease

**DOI:** 10.3389/fgene.2023.1359014

**Published:** 2024-01-05

**Authors:** Frontiers Production Office

**Affiliations:** Frontiers Media SA, Lausanne, Switzerland

**Keywords:** Alzheimer’s disease, educational attainment, intelligence, cognitive performance, Mendelian randomization, genome-wide association studies, conditional false discovery rate

Due to a production error, there was an omission of the indication of co-first authorship. Fuxu Wang, Haoyan Wang, Ye Yuan, and Bing Han are co-first authors.

In addition, the correct **Author contributions** statement is as follows, “FW, HW, YY, and BH have contributed equally to this work. FW, HW, YY, and SQ designed the study and analyzed the data. YH and TZ supervised the study. BH revised the manuscript. All authors contributed to the article and approved the submitted version.”

The publisher apologizes for this mistake. The original version of this article has been updated.

