# Supplementary material for: Integration of multiple-omics data to reveal the shared genetic architecture of educational attainment, intelligence, cognitive performance, and Alzheimer’s disease
Source: Front Genet. 2023 Oct 12;14:1243879. doi: 10.3389/fgene.2023.1243879 (PMC10601659; doi:10.3389/fgene.2023.1243879)

**Supplementary Figure 1.** Enriched gene ontology and pathways from shared risk genes of AD and educational attainment.


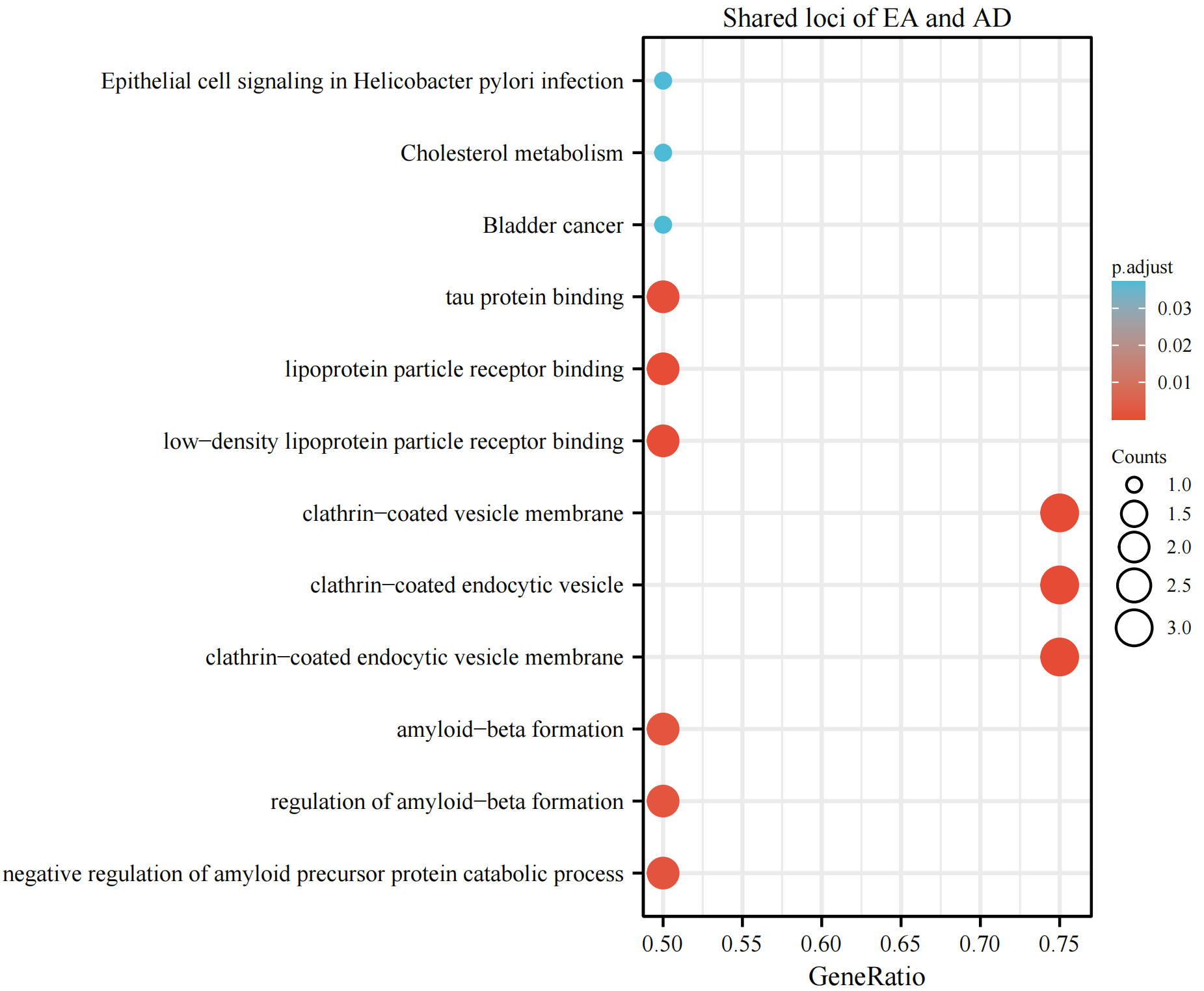


**Supplementary Figure 2.** Enriched gene ontology and pathways from shared risk genes of AD and cognitive performance.


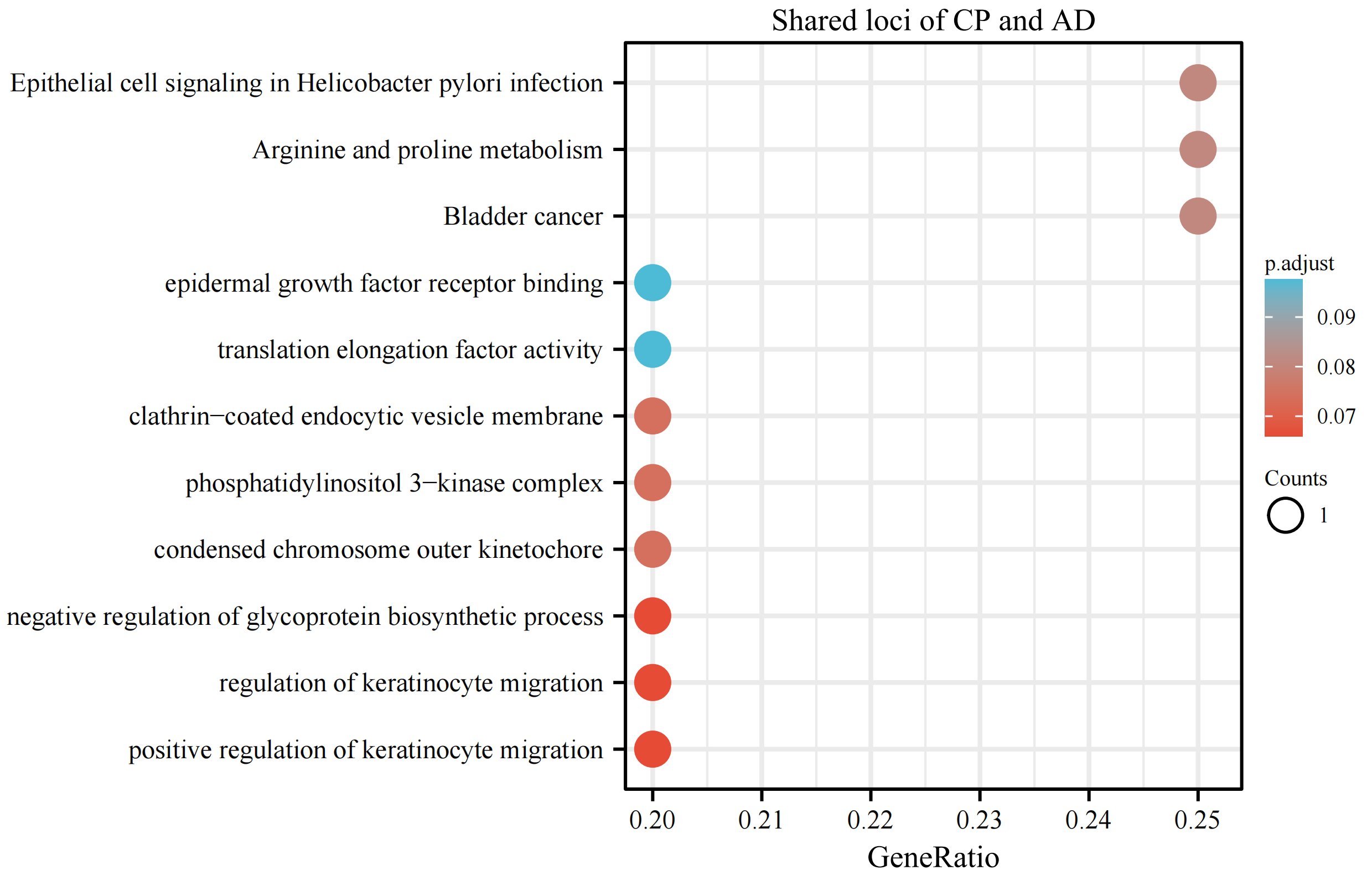

Supplement: Supplementary file 4 [file Table1.DOCX]
